# The Incidence and Risk Factors of De Novo Skin Cancer in the Liver Transplant Recipients

**Published:** 2012-11-01

**Authors:** J. Modaresi Esfeh, I.A. Hanouneh, D. Dalal, A. Tabba, R. Lopez, M. Pagadala, B. Eghtesad, N.N Zein

**Affiliations:** 1 *Department of Internal Medicine, *; 2 *Department of Gastroenterology and Hepatology, *; 3 *Department of Quantitative Health Sciences,*; 4 *Department of General Surgery, Transplant Center, The Cleveland Clinic, Cleveland, Ohio, USA.*

**Keywords:** Liver transplantation, *de novo* malignancy, skin cancer

## Abstract

**Background:** Liver transplantation (LT) increases the risk of *de novo* malignancies including skin cancers. However, risk factors for this type of cancers have not been well studied.

**Objective**: To determine the incidence of skin cancer in LT recipients, and to identify the risk factors of this type of cancer.

**Methods**: We identified all adult patients who underwent LT and developed *de novo* skin cancer post-LT at our institution between 1996 and 2009. We excluded the patients with history of skin cancer prior to LT. We also studied a control group of patients who underwent LT during the same period but did not develop skin cancer; the control group was matched (1:2) for age, gender and geographical place of residence.

**Results**: Over a median (IQR) follow-up of 41.5 (18.0, 98.6) months, 23 (2.3%) of 998 patients developed skin cancer post-LT, of whom 10 were identified with squamous cell carcinoma, 9 with basal cell carcinoma and 4 with melanoma. After adjusting the confounding variables, subjects who had combined liver/kidney transplant had 22 (95% CI: 5.1–99) times higher hazard of skin cancer compared to subjects with LT alone. Furthermore, patients who had non-skin cancer prior to LT had 23 (95% CI: 8.6–60) times higher hazard developing skin cancer after the transplant. Patients with history of alcohol consumption, as the underlying etiology of liver disease, had 4 (95% CI: 1.2–12.9) times higher hazard of developing skin cancer after transplantation. Type or duration of immunosuppression was not associated with increased risk of skin cancer post-LT. The post-LT survival outcome was not affected by the development of *de novo* skin cancer post-LT.

**Conclusion:** Skin cancer is relatively common in LT recipients and should be monitored, particularly in patients with a history of pretransplant malignancy, recipients of combined liver and kidney transplant or having alcoholic cirrhosis as the underlying cause of liver disease.

## INTRODUCTION

Liver transplantation (LT) is a life saving procedure for both acute and chronic liver failure [1]. In the new era of LT, the advances in the surgical techniques, the development of more efficacious immunosuppression and the improvement in ICU care in the peri-operative period, have improved both short- and long-term outcome post-LT [2]. However, with better post-transplant survival, patients face a new diversity of complications. *De novo* malignancies and cardiovascular events post-LT and recurrence of the underlying liver disease post-LT are some of the growing horizons in this field [3].

The risk of developing *de novo* malignancies post-LT is around 1% per year; with approximately 3% to 15% of liver transplant recipients develop new malignancy post-LT [4]. Moreover, *de novo* malignancy post-LT is the second leading cause of death post-LT, following cardiovascular complications [2, 3]. Skin cancer is the most common non-lymphoid malignancy observed among liver transplant recipients accounting for 6% to 20% of all cancers after transplant [5, 6-10]. Compared to skin cancer outside the transplant setting, the development of *de novo* skin cancer post-LT is characterized by higher incidence of multiple tumors in a single patient, and a younger age at the time of cancer diagnosis [5]. Additionally, while basal cell carcinoma is the predominant skin cancer in non-transplant population, squamous cell carcinoma is the most common *de novo* skin cancer in the LT recipients [5]. 

One of the main reasons for marked increase in the incidence of malignancy in the transplant population is the inhibition of the immune system by immunosuppressive agents used to prevent allograft rejection [11]. Bellnoch, *et al,* showed an increase in the rate of *de novo* malignancy at a shorter duration in more potent immunosuppressant protocols [12]. Several other factors may play a role in the evolution of *de novo* cancers post-LT. These include the genetic predisposition of donor and recipient, the age of the recipient at the time of transplant, potential oncogenic viruses (Epstein-Barr virus [EBV] and cytomegalovirus [CMV]), tobacco use and alcohol consumption. Identifying the risk factors of *de novo* cancer post-LT is of paramount importance for any strategy aims at improving outcome.

The objectives of the study were to determine the incidence of skin cancer in LT recipients and to identify the risk factors for this type of cancer.

## PATIENTS AND METHODS

After receiving an Institutional Review Board approval, we reviewed the electronic medical records of all adult patients aged ≥18 years who underwent LT at the Cleveland Clinic between January 1996 and December 2009 (n = 998). Patients who had definite diagnosis of skin cancer post-LT including basal cell carcinoma, squamous cell carcinoma and melanoma were identified. The diagnosis of skin cancer was based on the surgical pathology of the skin biopsy. Patients with history of skin cancer pre-transplant were excluded from the study.

A control group of patients who underwent LT during the same period and had no skin cancer post-transplant was identified. The two groups were matched (1:2 matching) for age, gender and geographical place of residence. 

Demographic characteristic of the donor and recipient including age, gender and ethnicity were collected. Moreover, the etiology of the underlying liver disease, history of malignancy pre-transplant, tobacco use and alcohol consumption were gathered from the transplant database of prospectively collected data. Post-transplant data included the type and the duration of immunosuppression used post-LT; number, severity and treatment of bouts of acute cellular rejection; and infection with CMV post-LT.

Immunosuppression

The main immunosuppressant agent used post-LT is calcineurin inhibitor. When side effects prevented administration of full therapeutic dose of calcineurin inhibitor, mycophenolate mofetil was added. Corticosteroids were discontinued 21 days after the surgery.

Mild episodes of acute cellular rejection were treated with increasing the trough level of calcineurin inhibitor, while moderate and severe rejections (defined as rejection activity index >5), were managed with intravenous corticosteroids.

CMV prophylaxis with seven-day course of intravenous valganciclovir was given for all cases with CMV-positive donors or CMV-positive recipients.

Statistical Analysis

A time-to-event analysis was performed to assess the factors associated with post-LT skin cancer. The length of follow-up was defined as the number of months between LT and the diagnosis of skin cancer or the last follow-up, if the patient remained cancer-free. Univariate and multivariate Cox regression analyses using a robust sandwich covariance matrix estimate to account for the intracluster dependence due to matching were used to analyze the data. An automated stepwise variable selection method performed on 1000 bootstrap samples was used to choose the final multivariable model; all factors were considered for inclusion; the four variables with the highest inclusion rates were included in the model. A p value <0.05 was considered statistically significant. All analyses were performed using SAS (ver 9.2, The SAS Institute, Cary, NC) and R (ver 2.13.1, The R Foundation for Statistical Computing, Vienna, Austria). Kaplan-Meier plots were constructed to compare the survival rate between patients who developed skin cancer post-LT and the control group.

## RESULTS

Patients’ characteristics

**Figure 1:**
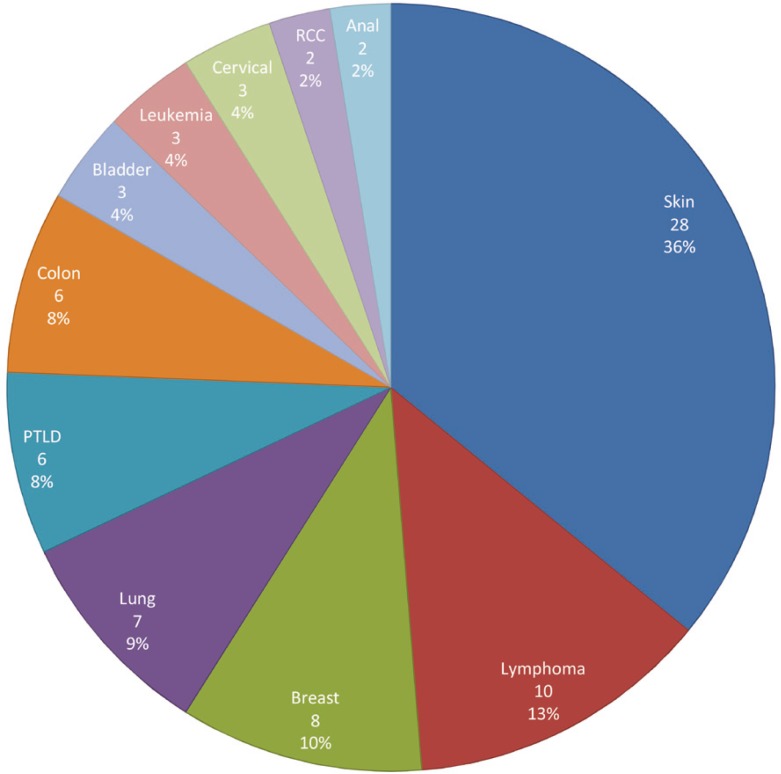
Type and rate of *de novo* cancer post-LT

A total of 998 liver transplant recipients was analyzed between January 1996 and December 2009 for the development of *de novo* cancers. Five patients were excluded from the study because they had a history of skin cancer before transplantation. Over a median (IQR) follow-up of 41.5 (18.0, 98.6) months, 75 (9.48%; 95% CI: 7.44%–11.53%) patients were identified with *de novo* cancer post-LT, of whom 23 (30%) with *de novo* skin cancer, and 52 (70%) patients with other malignancies post-LT. Figure 1 summarizes *de novo* malignancies observed post-LT in our study population.

A control group consisting of 46 patients who underwent LT during the same period and had no skin cancer post-transplantation was identified. The skin cancer and control groups were matched 1:2 for age, gender and geographical place of residence. The baseline characteristics of the 69 patients (23 cases and 46 controls) included in the study are summarized in Table 1.

**Table 1:** Post-LT skin cancer: Univariate Cox regression analysis

**Factor**	**No (%) post-LT skin cancer (n=46)**	**No (%) Post-Lt skin cancer (n=23)**	**Hazard Ratio (95% CI)**	**p value**
Male	32 (70)	16 (70)	1.08 (0.50–2.3)	0.85
Caucasian	33 (72)	23 (100)	∞	<0.001
Etiology*				
HCV	12 (27)	3 (13)	Ref	—
EtOH	5 (11)	3 (13)	4.7 (1.00–21.9)	0.048
EtOH/HCV	4 (9)	2 (9)	1.5 (0.37–6.0)	0.58
NASH	5 (11)	2 (9)	4.0 (0.87–18.5)	0.075
PSC/PBC	7 (16)	2 (9)	0.35 (0.04–2.8)	0.32
Cryptogentic	4 (9)	5 (22)	2.3 (0.44–12.1)	0.33
Other	8 (18)	6 (26)	4.1 (1.1–15.0)	0.035
OH Residence*	41 (91)	20 (87)	0.47 (0.13–1.7)	0.25
Cancer pre LT*	0 (0)	5 (23)	8.0 (3.3–19.1)	<0.001
Median (IQR) age at Tx	55.0 (47.0, 59.0)	57.0 (52.0, 59.0)	1.09 (1.00–1.2)	0.052
Transplant type*				
Liver	44 (98)	21 (91)	Ref	—
Kidney/Liver	1 (2)	2 (9)	11.8 (2.0–70.1)	0.007
MMF rx*	24 (63)	15 (65)	1.2 (0.55–2.7)	0.64
Cyclosporine rx*	6 (16)	3 (13)	0.69 (0.21–2.2)	0.53
Tacrolimus rx*	30 (81)	18 (78)	1.07 (0.38–3.0)	0.89
Sirolimus rx*	2 (5)	3 (14)	0.98 (0.39–2.5)	0.96
ACR*	14 (33)	4 (17)	0.39 (0.11–1.3)	0.13

CI: confidence interval

Etiology = 1, Resides in OH = 1, Cancer pre LT = 2, Transplant Type = 1, MMF rx = 8, Cyclosporine rx = 9, Tacrolimus rx = 9, Sirolimus rx = 10, ACR = 3

**The incidence of** ***de novo*** **skin cancer post-LT**

Among the patients with *de novo* skin cancers observed post-LT, there were 10 patients with squamous cell carcinoma (SCC), nine with basal cell carcinoma (BCC) and four with skin melanomas. This translates to an overall incidence rate of 84.8 skin cancer cases per 1000 person per year (SCC=36.9, BCC=33.2, and melanoma= 14.8 cases per 1000 person per year).

**Risk factors of** ***de novo*** **skin cancer post-LT**

History of malignancy pre-LT was associated with increased risk of developing skin cancer post-LT. Among patients with *de novo* skin cancer post-LT, there were five subjects with a diagnosis of malignancy prior to LT (three with hepatocellular carcinoma, single patient with lymphoma and one with transitional cell carcinoma of the bladder). Moreover, patients with combined liver/kidney transplant were more likely to develop skin cancer post-LT than those with liver transplant alone (p<0.001). Alcoholic liver disease also carries an increased risk of *de novo* skin cancer post-LT in our study population. Patients with alcoholic liver disease had four times greater risk of developing skin cancer compared to those with other forms of chronic liver disease (p=0.048).

Because all study patients with skin cancer post-LT were Caucasians, we could not examine the impact of ethnicity on the risk of *de novo* skin cancer post-LT. The age or gender of the recipient at the time of transplant was not associated with the risk of *de novo* skin cancer post-LT. Furthermore, the type or duration of immunosuppression, acute graft rejection and rejection activity index were not associated with increased risk of skin cancer post-LT.

After adjusting for confounding variables, three factors remained independently associated with the risk of *de novo* skin cancer post-LT. Subjects who had any sort of malignancy prior to transplantation had greater likelihood of developing skin cancer after the transplant (HR=23 [95% CI: 8.6–60]). In addition, compared to those who had a liver transplant only, people who had a combined liver/kidney transplant had 22 (95% CI: 5.1–99) times higher hazard of skin cancer post-LT. Lastly, patients with alcoholic liver disease were at 4 (95% CI: 1.2–12.9) times higher risk of *de novo* skin cancer post-LT compared to patients with other forms of chronic liver disease (Table 2).

Post-transplant survival outcome

**Figure 2:**
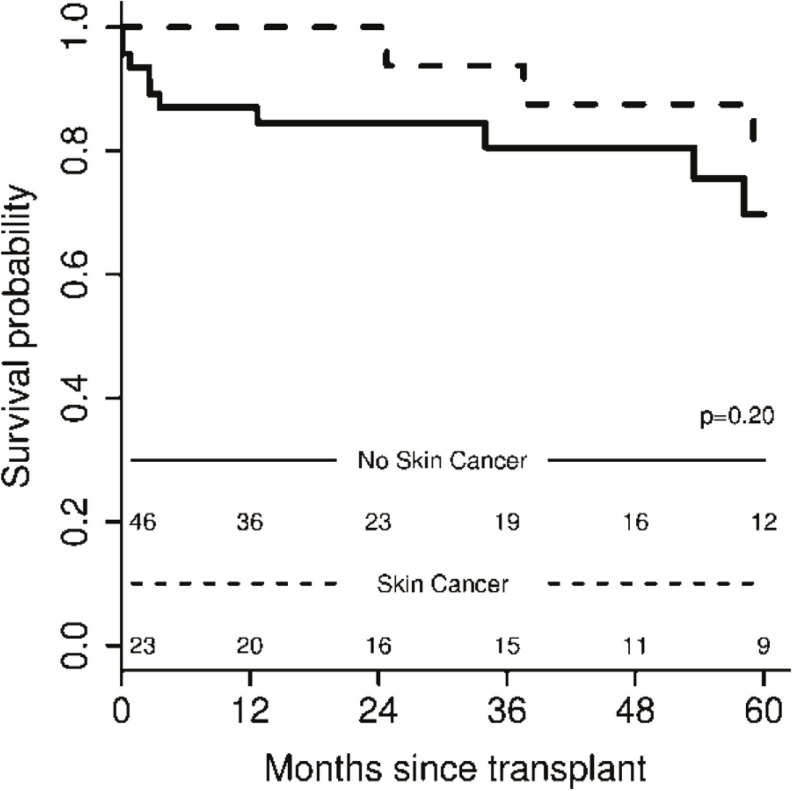
Post-LT survival in skin cancer and non-skin cancer liver recipients

The post-LT survival outcome was not affected by the development of *de novo* skin cancer post-LT (p = 0.2) (Fig. 2). Over a median (IQR) follow-up of 41.5 (18.0, 98.6) months, the post-LT survival outcome was estimated at 83% among patients with skin cancer post-LT, compared to 72% in patients who remained skin cancer free post-LT (p=0.32).

The survival outcome post-LT remained unaffected by *de novo* skin cancer post-LT after stratifying patients according to the type of skin malignancy (SCC *vs*. BCC *vs*. melanoma) (p = 0.16).

## DISCUSSION

LT is a life saving procedure for both acute and chronic liver failure. One of the long-term post-operative complication is *de novo* malignancy post-transplant. The incidence of *de novo* malignancies ranges from 3%–15% in different studies [13, 14]. Skin cancer is the most common non-lymphoid malignancy in the solid organ transplant [15]. The cumulative risk for *de novo* malignancy has been reported to increase the longer the allograft recipient survives, from 20% at 10 years to 55% at 15 years [13]. Our study showed an overall incidence rate of 84.8 skin cancer cases per 1000 person per year. These rates exceed what has been reported in other studies that have been done on post-LT population [13, 15]; the observed difference is likely due to the difference in the duration of follow-up.

The most common skin cancer post-LT in our study was SCC at 43% compared to 39% in BCC and 17% in melanoma. These results are consistent with several other studies that showed SCC is the most common type of skin cancer in liver recipients which is a reversal of the ratio 4:1 (BCC:SCC) observed in non-transplant population [13, 16].

In the past, most transplant centers were used to give either two or three medications for prevention of graft rejection in the immediate post-transplant period. This usually included a combination of a calcineurin inhibitor (cyclosporine or tacrolimus), a second agent like mycophenolate mofetil or azathioprine and finally, a glucocorticoid such as prednisone. Today, monotherapy with tacrolimus or cyclosporine in patients who achieve adequate liver function is the most popular maintenance regimen [17, 18]. There is controversy in the studies on the role of immunosuppressive agents in the development of *de novo* malignancy. It has been suggested that the type of the immunosuppression influence the incidence and type of *de novo* malignancy [19-21]. Mithofefer, *et al.*, showed a higher incidence of skin cancer in patients who were on cyclosporine comparing to tacrolimus [22]. However, this finding was not confirmed by other studies [17, 23-25]. We could not appreciate a statistically significant difference based on the type of immunosuppression (tacrolimus, cyclosporine, sirolimus and mycophenolate mofetil). Future studies with longer follow-up are needed to show if there is an association between the type of immunosuppression and skin cancer.

Another known risk factor for *de novo* malignancy is the underlying liver disease responsible for LT. Recipients with history of alcoholic cirrhosis as the underlying liver disease, are at greater risk of developing SCC of oral cavity, esophagus, pharynx, and larynx [4, 26]. In our study, alcohol as the etiology of liver disease was associated with higher risk of developing skin cancer post-LT.

Our study has a number of limitations. Firstly, this was a retrospective study with all its inherent biases. Secondly, it was shown in our study that pre-transplant malignancy and combined liver/kidney transplant increase the risk of *de novo* skin cancer post-LT; however, in this study there were five cases with pre-LT non-skin cancers and three with combined transplants. More studies should be done to confirm this association.

In conclusion, skin cancer is a common *de novo* malignancy in the post-LT recipients. Regular monitoring of the skin by both the patient and the physician should be considered. Simple steps including educating patients, advising regular use of sunscreens protection to avoid photo damage, monthly self skin examination and timely skin examination by a physician can be helpful in preventing and in the early detection of skin cancers post-LT.

## Notes

**Acknowledgments:** Mangesh Pagadala was supported by the grant number T32 DK061917.
